# Low temperature limits for root growth in alpine species are set by cell differentiation

**DOI:** 10.1093/aobpla/plx054

**Published:** 2017-10-19

**Authors:** Sebastian Nagelmüller, Erika Hiltbrunner, Christian Körner

**Affiliations:** Institute of Botany, Department of Environmental Sciences, University of Basel, Schönbeinstrasse 6, 4056 Basel, Switzerland; Institute of Agricultural Sciences, Swiss Federal Institute of Technology, Universitätsstrasse 2, 8092 Zürich, Switzerland

**Keywords:** Cell elongation, functional growth analysis, lignification; low temperature, root growth, sink–source relationships, xylem

## Abstract

Plant growth in cold climates is not limited by carbon assimilation (source activity) but rather by reduced carbon investment into new tissues (sink limitation). It has been hypothesized that all cold-adapted plants face similar growth constraints at low temperature mainly associated with the formation of new tissues. To explore the thermal limitation of plant tissue formation, we studied root growth and anatomical root tissue characteristics in four cold-adapted alpine species (*Ranunculus glacialis*, *Rumex alpinus*, *Tussilago farfara*, *Poa alpina*), grown in thermostated soils with a vertical temperature gradient approaching 1 °C. Above-ground plant organs were exposed to typical alpine climate conditions (high solar radiation and cool nights) at 2440 m a.s.l. in the Swiss Alps to assure continuous source activity. Image-based measurements of root growth (root elongation rates at 12-h intervals, RERs) were combined with anatomical examinations in thermally constrained root tips as well as with a functional growth analysis of entire plants. Temperatures in the range 0.8 to 1.4 °C were denoted as critically low temperature thresholds for root formation across the four species. The RERs per 12 h revealed that roots kept extending at low rates at 0.7–1.2 °C but cell elongation and xylem lignification were clearly inhibited in the terminal zones of root tips. Roots exposed to temperatures between 1 and 5 °C showed strongly reduced elongation rates so that these roots contributed very little to the entire root system compared to control roots grown at 10 °C. Hardly any secondary roots were formed at temperatures below 5 °C and total root mass was substantially lower (74 % reduction in comparison to control), also the above-ground biomass was reduced by 23 %. Cell elongation and differentiation rather than cell division control length and shape of root cells at the low temperature limit of growth. Lignification of root xylem is clearly constrained at temperatures below 3 °C.

## Teaser

This paper explores the causes of plant growth cessation at critically low temperatures in arctic-alpine environments. We grew four alpine plant species in thermostated soil cylinders in the field in the Swiss Alps, monitored root growth and studied root tip anatomy. Roots stopped growing at temperatures between 0.8 and 1.4 {degree sign}C. Microscopic examinations of root tips revealed that rates of cell elongation and differentiation control length growth. Xylem lignification appears to be a co-limiting factor at growth-limiting low temperatures.

## Introduction

In arctic–alpine environments, low temperatures constrain the growing season and thus biomass production of plants (Bliss 1956; Pollock and Eagles 1987; Körner 2003). Similar growth limitations by low temperatures were found for winter crops and plant species in grasslands (Gallagher 1979; Wingler and Hennessy 2016); therefore, it has been hypothesized that all cold-adapted plants underlie common growth constraints when temperature arrives at a critical threshold (Körner 2008). Above zero soil temperatures need to occur over at least 6 weeks for angiosperm survival (Körner 2011). Results of earlier works have indicated that tissue formation, irrespective of whether above- or below-ground, becomes very slow at or below 5 °C (Alvarez-Uria and Körner 2007; Körner 2008; Nagelmüller *et al.* 2016a) and was never observed at or below 0 °C, a temperature that still permits CO_2_ uptake at ca. 30 % of photosynthetic capacity. Hence, at such low temperatures plant growth is not carbon limited (Pollock *et al.* 1988; Xiong *et al.* 1999; Körner 2003, 2015). Similar low temperature thresholds were reported for leaf expansion as well as for root length increment (Körner and Woodward 1987; Schenker *et al.* 2014; Nagelmüller *et al.* 2016a), and radial growth of xylem (Rossi *et al.* 2007), suggesting that apical and lateral meristems exhibit similar temperature responses and face the same low temperature limitations at tissue and cell level. Leaves of cold-adapted Poaceae start expanding very slowly at close to 0 °C (Peacock 1975; Körner and Woodward 1987; Porter and Gawith 1999; Nagelmüller *et al.* 2016b). Although, absolute minimum temperature thresholds for growth do not explain the overall plant performance in cold climates, the analysis of tissue processes at such extreme thermal constraints provides insights into the underlying physiological and anatomical mechanisms that control life at the cold edge.

The production of new plant tissue includes cell division, cell enlargement and cell differentiation into various operational cell types (in that sequence). From what is known to date, cell division in cold-adapted plants is not interrupted at close to 0 °C (Francis and Barlow 1988; Körner and Pelaez Menendez-Riedl 1989). Cell enlargement depends on balanced rates of turgor-driven cell wall expansion and secondary cell wall synthesis. In graminoids, water flux into the vacuole (a major driver of cell expansion) does not appear to be affected over a temperature range from 2 to 20 °C (Thomas *et al.* 1989; Pollock *et al.* 1990). Even in a chilling-sensitive cucumber, vacuoles exposed to 8 °C had no problem to absorb water (Lee *et al.* 2005), and *Spinacia* plants rapidly adopted root hydraulic pressure after root temperature was reduced from 20 to 5 °C (Fennell and Markhart 1998). Hence, the critical processes are mainly to be associated with the growing cell wall. In the expansion zone of shoots and roots, cells undergo a several-fold size enlargement, which cannot be achieved with the initial primary wall. Secondary wall formation must go hand in hand with size increment; so, cell enlargement cannot be separated from differentiation, the most resource demanding process (Pollock and Eagle 1987). As part of that differentiation xylem and phloem become established. Xylogenesis notably contributes to the final biomass because of the thick xylem cell walls and their lignification. A low temperature-driven slowing of cell differentiation must feedback on cell division in order to retain mechanical robustness of the resulting tissue (Körner 2003). In conifers near the treeline, xylogenesis was found to cease at temperatures below 4–5 °C (Rossi *et al.* 2007, 2008). A lower temperature threshold for xylogenesis (2.0 ± 0.6 °C) was recently reported in the alpine shrub *Rhododendron aganniphum* (Li *et al.* 2016). However, we are dealing with an asymptotic decline, causing the absolute limit to become a matter of precision and definition. We suspect that cell differentiation (including lignification) is the most likely cause of root growth cessation at very low temperatures which otherwise still enable photosynthesis and cell division.

To explore these processes at tissue and cell level, we decided to use roots and root tips because roots grow in a thermally buffered environment, permitting to explore the effect of even minute temperature differences on meristematic activity at critically low (still positive) temperatures. Roots expanding from ambient soil surface temperatures towards critically cold conditions deeper in the soil allow identifying threshold temperatures and also to sample root tips developed under such cold conditions (Alvarez-Uria and Körner 2007; Schenker *et al.* 2014). We exposed four alpine plant species, *Ranunculus glacialis*, *Rumex alpinus*, *Tussilago farfara*, *Poa alpina*, to such conditions in the field. From prior research, employing cold glacier water runoff as cooling medium we delineated that the zero point for root growth is below 5 °C; however, a precise minimum temperature threshold could not been defined nor could the tissue level responses be assessed for the thermal limit of growth (Nagelmüller *et al.* 2016a). In the present study, we quantified anatomical/histological changes of cell expansion/differentiation in roots and root tips (root kinematics, Silk and Erickson 1979; Sharp *et al.* 2004) grown at precisely controlled temperatures below 3 °C in order to identify the absolute minimum temperature threshold for root growth and cell elongation and differentiation in alpine plants. We expected a continuous cell division but a delay in the rate of cell enlargement and cell differentiation, causing this zone of the root tip to lengthen relative to controls at 10 °C. We also anticipated a weaker lignification, hence, a longer stretch of poorly lignified tissue behind the root tip as it reaches its low temperature growth limit.

## Methods

### Experimental setup

The experiment was conducted at the ALPFOR research station, close to the Furka Pass, at 2440 m a.s.l. in the Swiss central Alps. Individuals of four alpine plant species, *R. glacialis* (Ranunculaceae), *R. alpinus* (Polygonaceae), *T. farfara* (Asteraceae) and the grass species *P. alpina* ssp. *vivipara* (Poaceae), were collected at a very early seasonal developmental stage. We selected plantlets with newly emerging root tips of <2 mm length on the day of sampling. Roots from the previous growing season were cut to 3 cm length for later distinction from newly developed roots. For each species, we planted 42 individuals in cylindrical containers so that the apical meristem was positioned at −1 cm soil depth, which also correspond to the position of the youngest newly emerging root tips.

**Figure 1.**
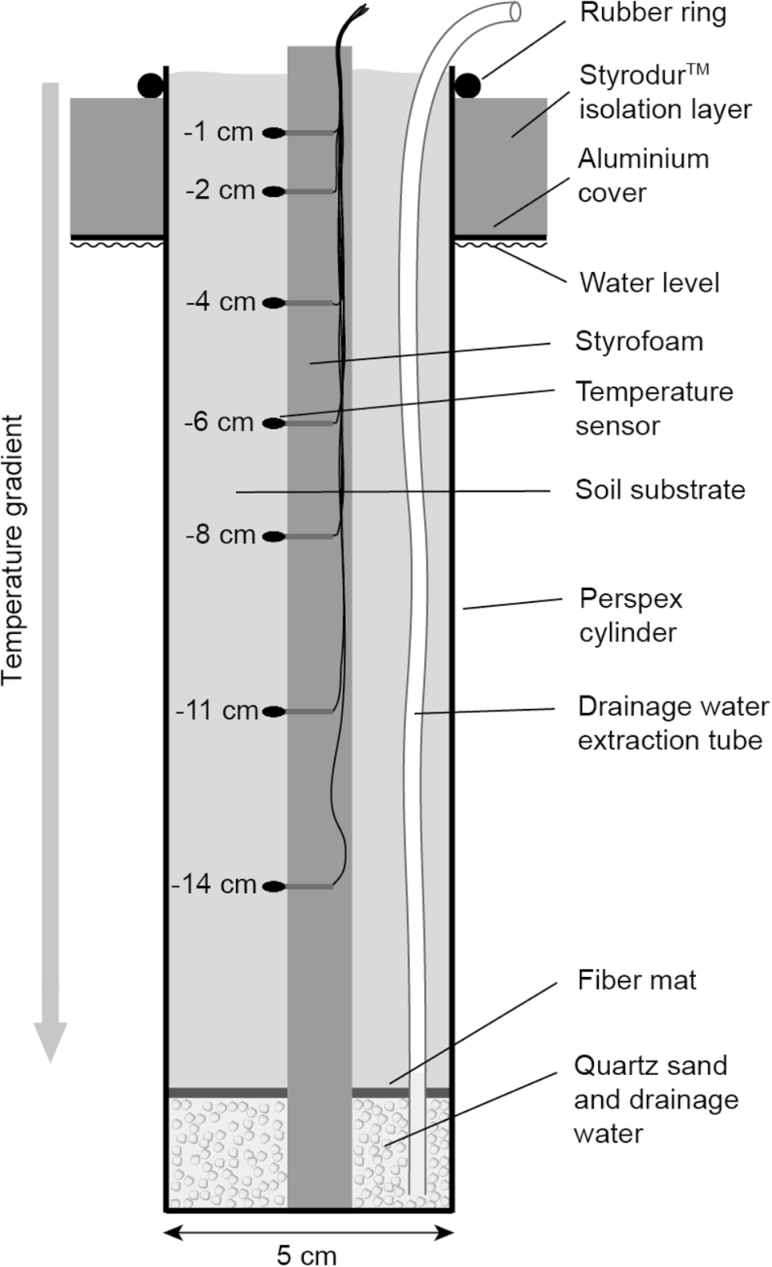
Design of a plant cylinder with temperature sensors placed at different soil depth.

Half of the plants were planted in Plexiglas® cylinders (200 × 50 mm, 1 mm wall thickness, Evonik Industries, Essen, Germany), which allowed image-based root elongation measurements. The other half was planted in correspondingly sized polypropylene tubes with 0.8 mm wall thickness (‘p-Safe PP’, 5-P KG, Sulz, Germany), appropriate for measuring final rooting depth and the final harvest of root tips as well as the total root biomass. Both types of cylinders had a watertight seal at the bottom. The lowest 2 cm of each cylinder were filled with quartz sand (grain size: 2 mm) for drainage water, separated from the growth substrate by a fibre mat (Fig. 1). The upper 18 cm of the tube length was filled with a substrate mixture of 80 % fine sandy glacier silt and 20 % potting compost (Capito Universalerde, Fenaco, Bern, Switzerland). To exclude any growth-limiting factor except temperature in such an artificial growth substrate, fertilizer was provided weekly (in total three times over the 29-day experimental period) by adding 10 mL full strength Hoagland’s solution (1.6 g L^−1^ of Hoagland salts; Sigma-Aldrich, Munich, Germany). Plants were watered with 20 mL water every second day in case there was no precipitation. Excess water (also from rain) drained to the bottom, was removed with a hand pump, using a 3 mm tube that reached the cylinder bottom.

Plants were exposed for 29 days during the main part of the growing season to have low and high substrate temperatures by immersing the cylinders into four double-walled 96 L stainless steel, thermostated water tanks (interior dimensions: 80 × 60 × 20 cm). Three of these water baths were set to 1 °C and one to 10 °C as a control, resulting in temperatures of ca. 1.5 and 10.3 °C of the circulating cooling water (see Results section). Thirty plant replicates per species were placed in each of the low temperature baths, and 12 plant replicates per species were placed in the ‘warm’ control bath. Cylinders were randomly arranged in the water baths.

**Figure 2.**
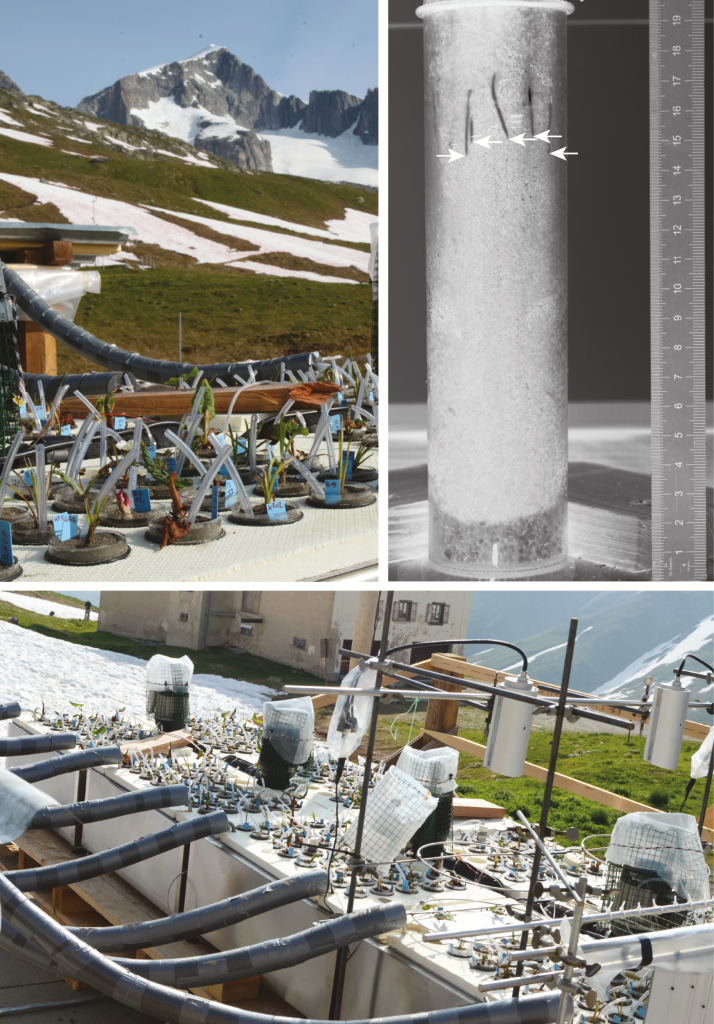
Thermostated water bath systems at the Furka Pass (2440 m a.s.l.) with transparent cylinders for root observation (measurements of RERs). The black to white converted image of a root cylinder taken in a photo box shows roots of *Rumex alpinus* growing along the wall. White arrows point at root tip positions.

Each water bath was equipped with a thermostat system (CBN 28-30 and HTM 200, Heto-Holten, Allerød, Denmark) and a water-circulating system to ensure uniform temperature distribution in the water baths. To minimize vertical heat flow, water baths were covered by a 2 mm aluminium plate and with a 2 cm Styrodur™ isolation layer on top. These covers had 50 mm diameter holes into which 40 cylinders per bath were inserted and fastened by a rubber ring (Fig. 1). The rings also prevented light from leaking into the ‘below-ground’ compartment. The water bath systems were placed on the terrace of the ALPFOR station to expose above-ground plant organs to typical alpine climate conditions (Fig. 2; **see Supporting Information—Fig. S2**).

The soil temperature gradient was measured in two cylinders per water bath (named T-cylinders) each equipped with seven small temperature sensors (NTC-resistors, 2 mm in diameter, 5 kΩ at 25 °C, ±0.2 K, Epcos, Munich, Germany) at depths from −10 to −140 mm in the cylinders (Fig. 1) and with 1 cm distance to the cylinder wall. Plants were absent in these T-cylinders. Water temperatures in each water bath were measured by another set of NTC-resistors close to the water-circulating system. Temperatures in T-cylinders were recorded every 10 min with a data logger (CR1000, Campbell Scientific, Logan, UT, USA) and two AM16/32B multiplexers (Campbell Scientific). Hourly temperature means were further used for all calculations.

Due to short periods of equipment failure early in the experiment and not of noticeable impact on the root elogation rate (RER) or the harvest data, the water in the cold treatment bath 3 heated up to 19 °C for 14 h on treatment-day-3 and up to 9 °C for 7 h on treatment-day-9 **[see Supporting Information—Fig. S1]**. In the warm control treatment, water heated up to 21 °C for 12 h on treatment-day-1. The brief temperature deviations emerged before RER measurements started, and except for the 7 h on Day 9, occurred immediately after planting, when plants were still affected by the transplantation.

### Growth measurements

RERs were obtained for a period of 9 days and started on treatment-day-14 when sufficient roots arrived at the transparent cylinder walls of the cold treatment. Digital images (25 pixel mm^−1^) of the Plexiglas® cylinders were taken in 12-h intervals (0700 in the morning and 1900 CET in the evening) by using a photo box (100 × 60 × 60 cm) equipped with a digital camera (Nikon D7000, Nikon, Tokyo, Japan) with a 35 mm lens (Nikon) and a flash (64AF1, Metz, Zirndorf, Germany) for illumination. To ensure consistent image frames, cylinders were placed at exactly the same distance and orientation using positioning guides. Collecting and repositioning cylinders and taking photos took <90 s.

The position of root tips was tracked across the sequence of images by using the software ‘ImageJ’ (version 1.47v, Rasband 1997–2015) and the plug-in ‘SmartRoot’ (Lobet *et al.* 2011). We measured root length increment to calculate RERs (RER mm per 12 h) and the root tip position in the cylinders (soil depth). The root tip positions were related to the temperature profiles along the cylinders **[see Supporting Information—Fig. S3]**.

All plant individuals were harvested after 29 treatment days, which represents the main part of the alpine growing season. Both types of cylinders were opened at the bottom. The substrate was carefully removed until the tip of the single, deepest root became visible (without stretching the root) and maximum rooting depth was measured with a ruler. These depth values of roots reaching the coldest soil layers (averaged from several single deepest roots of cylinders) were used to calculate the mean minimum temperature threshold for root growth for each species. Thereafter, plants were washed and photographed and separated into roots, leaves and stems including flowers. Leaves and roots were scanned with a transmitting light scanner (Epson Expression 1680, Epson, Meerbusch, Germany). Total root length, numbers of primary and lateral roots were calculated from scans, using the WinRHIZO software (Regent Instruments Inc., Quebec, Canada). The dry weight of roots, leaves and stems was determined after drying at 80 °C for at least 48 h. Specific root length (SRL) and specific leaf area (SLA) were calculated by dividing the total root length (m) and leaf area (cm^2^) by the corresponding dry weights (g). For biomass allocation (functional growth analysis), mass fractions (leaf, stem and root mass fractions) were calculated by dividing the dry weights of the fractions by the total plant weight.

### Root anatomy

Longitudinal thin sections (tangential) with 80 µm thickness of the 2 cm terminal part of the root including the tip were made through the central cylinder in order to assess (i) cell density per unit root area, (ii) cell length in the elongation zone and (iii) cell differentiation (lignification) as described below. We sampled root tips from several single deepest roots of different cylinder at the day of harvest and stored them in 75 % (v/v) ethanol. Root tips were cut in pieces of 5 mm length, embedded in 3 % (w/v) agarose gel and cut by a vibratome (VT1200, Leica Biosystems, Nussloch, Germany). To visualize lignification in the xylem, thin sections of 80 µm were stained following Brundrett *et al.* (1988) starting with 1 h in 0.1 % (w/v) berberine hemisulphate followed by 30 min in 0.5 % (w/v) aniline blue at room temperature. Although berberine is not considered a lignin-specific dye (Brundrett *et al.* 1988), it intensifies the fluorescent signal of lignified xylem cell walls in contrast to non-lignified walls and allowed us to quantify the lignification optically. In addition, counter-staining with aniline blue inhibited any other fluorescence signals in the root tissues. Stained sections were mounted on microscope slides in 50 % (v/v) glycerine with 0.1 % (w/v) of FeCl_3_ as a preservative. Sections were viewed with a fluorescence microscope (Leica DM 2500, Leica Microsystems, Wetzlar, Germany) equipped with an UV-filter set (excitation filter BP 320-280 nm, chromatic beam splitter FT 400 (400 nm), emission filter LP 425 nm). For image analysis, series of overlapping images were taken along the root by a digital microscope camera (Leica DFC 300 FX, 3.2 pixels mm^−1^) with constant 100 ms exposure time. Single images were merged by eye with the program ‘Illustrator CS5’ (Adobe Systems Incorporated, San Jose, CA, USA) to display a longitudinal section of the entire 20 mm root tip.

We (i) counted the number of cells in a 0.2 × 0.2 mm square which was positioned as close as possible to the root meristem’s initials (root apex, beneath the root cap), determined (ii) the mean final cell length in roots, measured (iii) the distance from the root apex to the position at which final cell length was reached to define the length of the cell elongation zone and (iv) assessed the degree of lignification of the xylem along the root. For (iv), we selected four image snippets (0.6 mm in diameter) of the central cylinder every 4 mm along the root starting at the root apex. Pixels of image sections were examined for lignification over a defined strip of 10 pixel width (ca. 0.2 mm) over the length of the four snippets using MATLAB 8.2 (The Mathworks, Natick, MA, USA). Then, we extracted the L-channel values of the HSL colour space (H = hue, S = saturation and L = index of lightness, with values between 0 and 1, RGB red-green-blue colour model) of the selected pixels and averaged these values by the number of pixels. These light intensity indices were used as a quantitative proxy for lignification. In addition, we measured (v) the distance from the root apex to the first lignified (fluorescent) xylem element. Cell size and distance measurements were done using the program ‘ImageJ’ (see above).

### Data analysis and statistics

We calculated the correlations between soil temperature and soil depth for each water bath and the measured RERs during 12 h (mm per 12 h) as well as the root tip positions at harvest using third-order polynomial regressions as the temperature decrease between −20 and −80 mm soil depth was non-linear. Root tip temperatures were then estimated using the polynomial functions, based on the root tip positions at the end of each 12-h interval. Hourly temperatures within each RER 12-h interval were used for the polynomial fit as well as hourly minimum and maximum temperatures during the 12-h interval were considered **[see Supporting Information—Fig. S3]**. Since root tips occurred over a narrow range of the profile only (with curve fitting outside that range not relevant for root tips), we also estimated root tip temperatures derived from two combined linear regressions between the sensor depths −20 to −40 mm and −40 to −60 mm, where most cold-treated roots grew. These temperatures differed from temperatures from the polynomial regressions on average by ±0.017 K (SD) with a maximum deviation of 0.02 K. Since this deviation is below the T-sensor accuracy, we are confident that the polynomial regressions reflect the root tip temperature in the root observation window with the needed precision. To analyse the relationship between RERs (mm per 12 h) and the root tip temperatures, linear models were applied. The minimum temperature for root growth was derived from the single deepest root within a cylinder reached at the day of harvest (after 29 full treatment days). Here, we used for the polynomial fit of all hourly temperatures after the last RER measurement until the root harvest. We also calculated a mean by averaging the deepest root tip positions of several cylinders per species and the corresponding temperatures at these positions.

To test for differences between temperature treatments, we performed one-way ANOVAs for: RER, total root length, root dry weight, SRL, number of primary and secondary roots, below-ground biomass (BGB), leaf area, leaf mass, SLA, above-ground biomass (AGB), biomass fractions, the lengths of the root elongation zone and the distance from root tip where first lignified xylem was detected. Additionally, we performed a linear model to test for differences in light intensity of the fluorescent xylem between temperature treatments plus for the light intensity increase along the root length (nested design) for each species. For the analysis of the post-harvest data, we merged the data from the three cold water baths since no significant differences we found among water baths (n.s. for factor bath). Number of replicates for different traits often deviated from the number of replicates at the beginning of the experiment, since individuals varied in the performance of certain traits. The normal distribution of post-harvest data was tested visually (q-q plots, histograms) and as log-transformation did not yield different statistical outcomes, non-transformed data are here presented. All statistical analyses and diagrams were done with R Statistical Software (version 3.0.2; R Development Core Team 2014) and the package ‘ggplot2’ (Wickham 2009).

## Results

### Soil temperature

**Figure 3.**
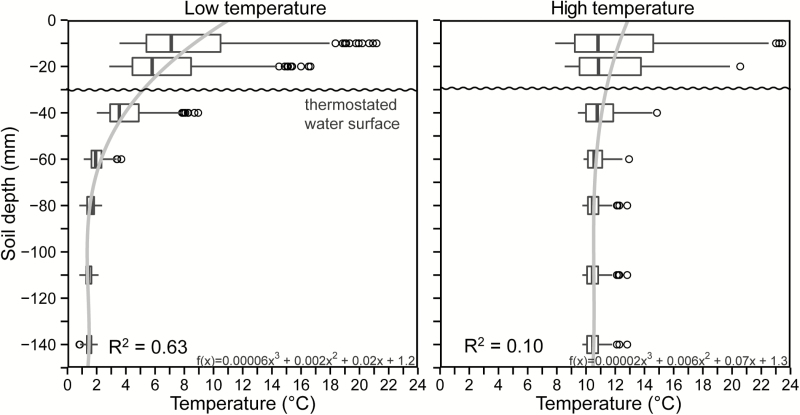
Polynomial temperature profiles (grey line) in cold-treated (left) and warm-treated (right) plant cylinders calculated during measurements of RERs. Data of the three cold water baths were averaged. Boxplots display the hourly temperature readings for each sensor depth. Boxes comprise the 25–75 % quantiles with the median as vertical line, dots indicate outliers. The deepest temperature sensor was located at −140 mm depth. Similar polynomial regressions were made for each RER 12 h^−1^ to estimate root tip temperatures.

The cooling water bath systems provided a hourly mean of water temperatures of 1.5 ± 0.4 °C (±SD) in bath 1, 1.5 ± 0.6 °C in bath 2, 1.4 ± 0.9 in bath 3 during the experiment, underpinning that the temperature regime among the three baths was identical. The mean temperature for all hourly intervals of the warm water bath was 10.6 ± 0.5 °C. The temperature in the cylinders declined with soil depth in both treatments. In the cold treatment, this temperature decrease was particularly pronounced between −10 and −80 mm (*R*^2^ = 0.96, *P* <0.001, Fig. 3) and most cold-treated roots grew not deeper than −80 mm. Temperature decreased from a hourly mean of 8.5 ± 4.3 °C at the top (−10 mm, ±SD) to 1.5 ± 0.8 °C at −80 mm depth and the temperature further approached 1 °C below −80 mm soil depth. In the warm treatment, the temperature gradient was less steep, ranging from a hourly mean of 12.2 ± 3.9 °C at −10 mm to 10.6 ± 0.5 °C at −140 mm (*R*^2^ = 0.72 ± 0.23, *P* < 0.001, Fig. 3). The diurnal temperature fluctuations in the upper 40 mm of the soil column were caused by fluctuating solar radiation in both, cold and warm treatments, especially, since the uppermost 30 mm of the cylinders were not immersed in the water but insulated by the Styrodur layer. At −60 mm soil depth, these fluctuations became minor and the temperature differences of 8–10 K between the cold and warm treatment were stable (Fig. 3; **see Supporting Information—Fig. S1**). In the deepest layers where roots still grew, the hourly maximum temperatures never surpassed 5.2 °C at −60 mm, 2.2 °C at −80 mm and −110 mm soil depth in each of the three cold water baths.

### Root length increment and temperature

**Figure 4.**
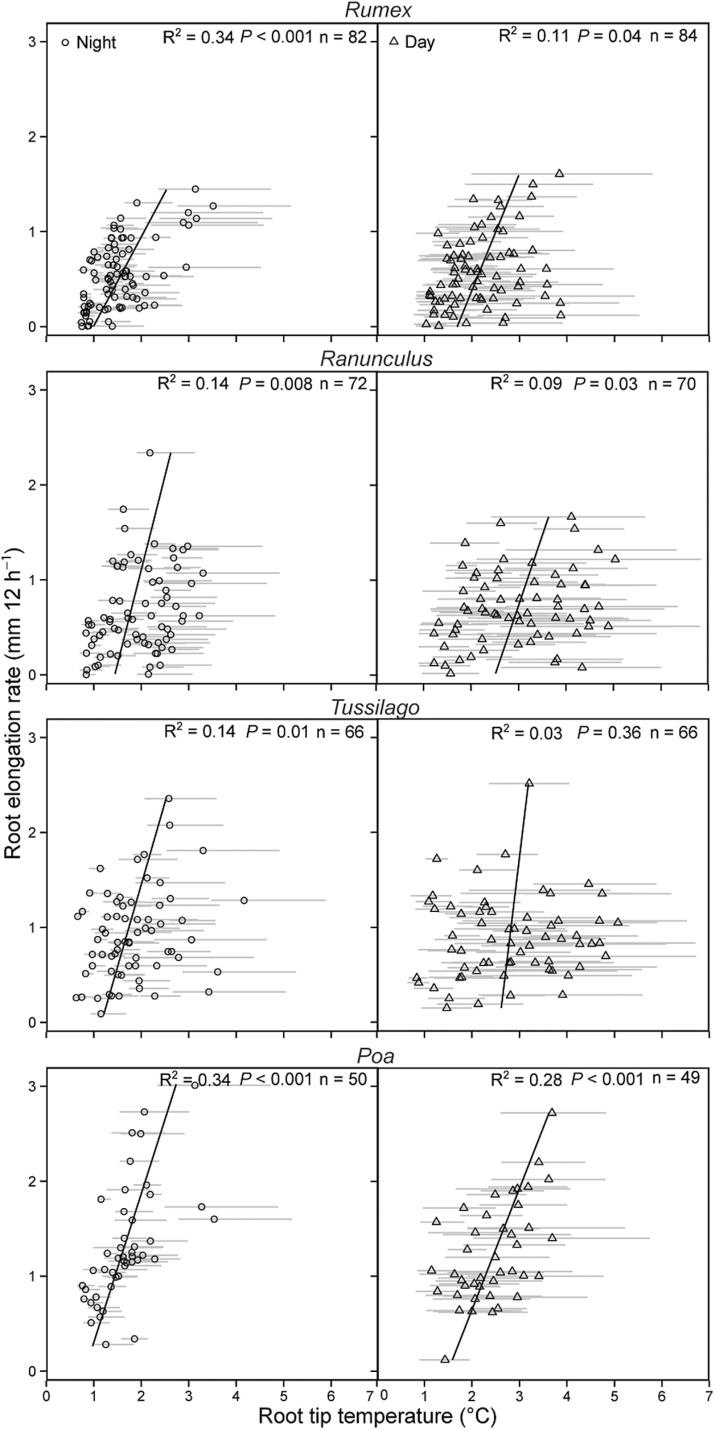
RERs during 12-h intervals (right: day; left: night) in relation to temperature at root tip position (cold treatment only). Each dot presents the mean temperature and the line the range between hourly minimum and maximum temperatures during the 12-h intervals. Number of replicates: *Rumex*: *n* = 10 roots of 10 different individuals; *Ranunculus*: *n* = 9; *Tussilago*: *n* = 8; *Poa*: *n* = 7.

RERs per 12-h interval in the cold treatment were linearly and positively correlated with the mean root tip temperatures (mean of 12 root tip temperatures during the corresponding interval) in all four species. Within each species, linear regressions between RER (mm per 12 h) at night and root tip temperatures were always closer than regressions with RER mm per 12 h day values (Fig. 4). For each mean root tip temperature, we also presented the coldest and warmest hour during the 12-h interval (grey line in Fig. 4). Especially during the night interval, the warmest hour did not affect the mean root tip temperature, indicating by the skewed position of the mean on the grey line.

We were able to record very small RERs of <0.2 mm per 12 h of a few individual roots between 0.7 and 1.2 °C during overcast days and cooler night intervals at ca. −60 mm soil depth (Fig. 4). However, comparing the RERs at these low temperatures between the four species indicates that species may vary substantially in their capability to elongate their roots at these low temperatures. For instance, *Poa* roots elongate 0.6 mm per 12 h, whereas the RERs of the three forbs were lower (Table 1).

**Table 1.** Root length responses near the low temperature limit. RERs during the 12-h interval with the lowest temperature in the cold treatment, the deepest single root tip position and the mean maximum root tip depths (averaged across cylinders per species ± SD) at harvest. Corresponding root tip temperatures were derived from the temperature gradients (polynomial functions, calculated for 12 h for RER and for the last five treatment days prior to harvest). ^#^Hourly minimum and maximum temperatures for *n* = 26–30 single roots per species during root formation.

	*Rumex*	*Ranunculus*	*Tussilago*	*Poa*
RER (mm per 12 h)	0.4	0.2	0.3	0.6
Temperature (°C) during 12-h interval (hourly min–max per 12 h)	0.9 (0.8–1.1)	0.9 (0.7–1.2)	0.7 (0.6–1.1)	0.7 (0.7–1.0)
Single deepest root tip position (mm)	73.7	77.3	87.6	105.8
Root tip temperature (°C; hourly min–max)	1.2 (1.1–1.4)	1.0 (0.8–1.2)	1.0 (0.9–1.3)	1.0 (0.8–1.3)
Mean of maximum root tip positions (mm; ±SD)	54.4 ± 11.9	61.5 ± 12.3	65.5 ± 14.5	91.7 ± 13.1
Number of deepest root tips (*n*)	*n* = 26	*n* = 30	*n* = 26	*n* = 29
Mean of root tip temperatures (°C; ±SD):	2.4 ± 1.1	2.0 ± 0.9	1.9 ± 0.8	1.5 ± 0.3
Hourly min/max temperatures^#^	1.1/5.3	0.8/5.1	0.9/4.7	0.8/2.8

As expected, RERs between 1.0 and 5 °C were always significantly lower than the RERs in the control (*P* < 0.001 for all species, Table 2). No correlation was found between RERs and the small variation in root tip temperature in the warm control (data not shown).

**Table 2.** RER, root traits, shoot traits and biomass fractions (mean ± SD). *P*-values from one-way ANOVAs, testing trait as dependent variable between cold and warm treatment for each species. Significance differences at ***P < 0.001; **P < 0.01; *P < 0.05.

	*Rumex*			*Ranunculus*			*Tussilago*			*Poa*		
	Cold	Warm	*P*	Cold	Warm	*P*	Cold	Warm	*P*	Cold	Warm	*P*
Below-ground												
RER (mm per 12 h)	0.5 ± 0.4	4.4 ± 1.6	***	0.7 ± 0.4	2.5 ± 0.9	***	0.9 ± 0.5	3.2 ± 1.1	***	1.2 ± 0.5	3.5 ± 1.4	***
Total root length (cm)	26.6 ± 15.8	858 ± 267	***	19.6 ± 10.6	153 ± 66	***	42.4 ± 20.9	478 ± 223	***	46 ± 31	477 ± 198	***
Root dry weight (g)	0.01 ± 0.01	0.17 ± 0.06	***	0.03 ± 0.02	0.09 ± 0.04	***	0.02 ± 0.02	0.08 ± 0.04	***	0.02 ± 0.01	0.05 ± 0.02	***
SRL (m g^−1^)	19.1 ± 4.3	52.8 ± 10.3	***	8.31 ± 3.7	19.6 ± 7.9	***	23.5 ± 8.6	58.6 ± 7.2	***	27.8 ± 10.5	100 ± 22.6	***
No. of primary roots	9.4 ± 4.2	12 ± 2.8	0.06	5.6 ± 2.1	5.9 ± 1.8	0.71	8.7 ± 2.9	11.7 ± 5.8	*	5.3 ± 2.4	9.8 ± 3.1	***
No. of secondary roots	7 ± 8.1	1959 ± 557	***	6.7 ± 10.5	176 ± 73	***	19.2 ± 17.1	829 ± 436	***	33.7 ± 30.7	1344 ± 536	***
BGB (g)	0.87 ± 0.31	1.06 ± 0.29	0.08	0.09 ± 0.03	0.12 ± 0.05	*	0.14 ± 0.05	0.19 ± 0.04	**	0.08 ± 0.02	0.12 ± 0.03	***
Above-ground												
Leaf area (cm^2^)	24.9 ± 7.95	74.8 ± 14.3	***	4.72 ± 1.89	5.86 ± 1.82	0.09	7.19 ± 1.87	19.3 ± 8.51	***	3.77 ± 1.29	8.33 ± 2.27	***
Leaf mass (g)	0.23 ± 0.07	0.39 ± 0.09	***	0.05 ± 0.02	0.06 ± 0.02	0.17	0.07 ± 0.02	0.12 ± 0.05	***	0.04 ± 0.02	0.06 ± 0.03	*
SLA (m^2^ g^−1^)	11.4 ± 3.53	19.9 ± 2.97	***	9.44 ± 1.78	9.41 ± 0.93	0.95	10.9 ± 1.31	15.5 ± 2.14	***	9.09 ± 2.57	16.6 ± 9.79	***
AGB (g)	0.32 ± 0.11	0.52 ± 0.13	***	0.13 ± 0.05	0.11 ± 0.03	0.23	0.09 ± 0.03	0.16 ± 0.06	***	0.11 ± 0.04	0.15 ± 0.05	**
Biomass fractions												
LMF	0.68 ± 0.05	0.56 ± 0.05	0.12	0.33 ± 0.06	0.33 ± 0.08	0.23	0.57 ± 0.07	0.52 ± 0.05	0.23	0.38 ± 0.13	0.31 ± 0.05	0.44
SMF	0.25 ± 0.04	0.19 ± 0.02	0.07	0.49 ± 0.11	0.24 ± 0.09	**	0.26 ± 0.06	0.14 ± 0.03	**	0.47 ± 0.12	0.45 ± 0.06	0.62
RMF	0.04 ± 0.02	0.24 ± 0.07	***	0.16 ± 0.08	0.42 ± 0.11	***	0.17 ± 0.08	0.33 ± 0.05	***	0.14 ± 0.05	0.24 ± 0.03	**

Root length increments in the cold treatment became smaller with increasing soil depth (data not shown) but roots still continued elongating very slowly as they approached the low temperature RER threshold.

At harvest the temperatures at the maximum rooting depth obtained, were in the range of 0.8 to 1.4 °C for all four species, taking the hourly maximum temperatures during the root-forming period into account (Table 1). In *Poa*, the single deepest root grew to 106 mm corresponding to a temperature of 1.0 °C with hourly minimum and maximum temperatures of 0.8 and 1.3 °C, whereas the deepest roots of the other species were found between 74 and 87 mm (Table 1). Averaging the deepest single roots across several cylinders supported the difference between the three forbs and the grass species. *Rumex* roots stopped at higher soil depth, thus slightly warmer temperature, corresponding to 2.4 ± 1.1 °C, then *Ranunculus* at 2.0 ± 0.9 °C, *Tussilago* at 1.9 ± 0.8 °C and *Poa* at 1.5 ± 0.3 °C (Table 1). The roots of the 10 °C control all reached the bottom of the cylinders (180 mm).

### Root, leaf and plant traits

At harvest (after 29 full treatment days) total root length of cold-treated plants, including first- and second-order roots, reached only 3 % (*Rumex*), 9 % (*Ranunculus*), 13 % (*Tussilago*) and 10 % (*Poa*) of the length of roots of the control plants (Table 2). Final root dry weight was similarly affected. In cold soils, the root systems were not only reduced in size (Table 2), but roots were significantly lighter per unit length that means reduced SRL m g^−1^ in all species. Low temperature almost completely inhibited the development of lateral roots, compared to the high number of secondary roots in the warm treatment (Table 2). If any, lateral roots were found only close to the root base in the warmer uppermost centimetre of the substrate (data not shown). The root mass fraction (RMF) was significantly lower in the cold substrate in all species (−66 % across all species in comparison to the controls).

Leaf area (cm^2^), leaf dry mass (g), SLA (m^2^ g^−1^) and also the total above-ground dry weight (g) were significantly smaller and lower, respectively, in cold compared to warm soils except for *Ranunculus*. *Ranunculus* showed a slightly reduced leaf area in the cold but the leaf mass, SLA and total above-ground dry weight did not differ between the temperature treatments. Leaf mass fraction (LMF) was not affected by the temperature treatments. Unexpectedly, stem mass fraction (SMF) was higher in *Ranunculus* and *Tussilag*o under cold treatment but slightly lower in *Rumex* and unaffected in *Poa* (Table 2).

### Root anatomy

For each species, the deepest roots were selected for the anatomical assays. Nevertheless, the number of root replicates per species dropped as only perfectly longitudinal sections of roots were further processed (*n* = 5 for *Poa* and *Ranunculus*; *n* = 7 for *Rumex* and *Tussilago*). For *Poa*, the hourly maximum root tip temperatures of the selected roots were never higher than 2 °C, for *Rumex* (except one root), *Ranunculus* and *Tussilago* (except one root), root temperatures never surpassed 3 °C.

**Figure 5.**
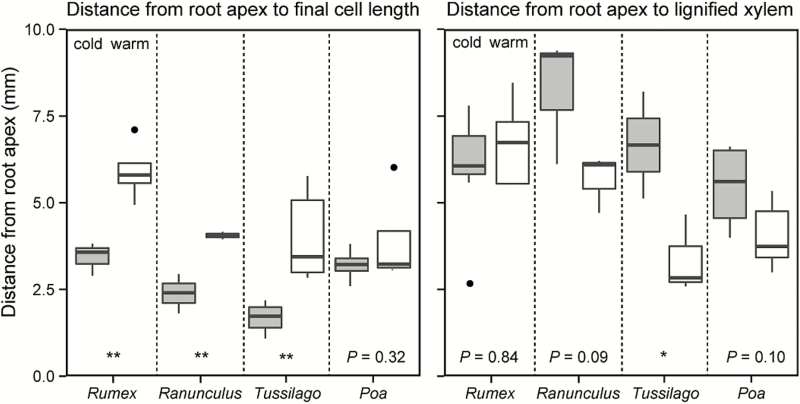
The distance between the root apex and the first cells reaching final cell length (left), and the distance between the root apex and the first lignified xylem elements (right; for number of replicates, see Table 3).

**Figure 6.**
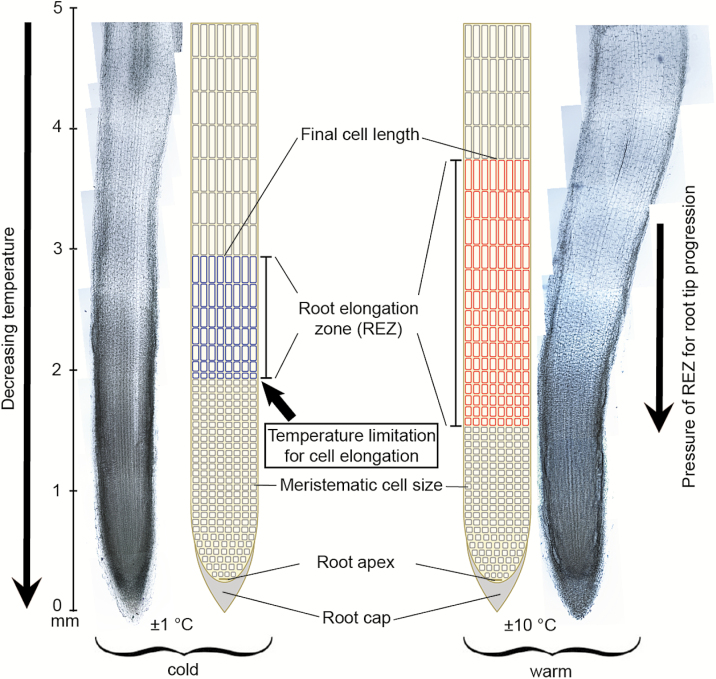
Root tips (5 mm length) of *Tussilago farfara* are shown as an example to visualize the effect of a shortened root elongation zone under low temperature. Due to the vertically decreasing temperature, cell elongation to final length was inhibited when roots reached a certain soil depth, which means lower temperature.

The cell density in the root tip, counted right after the root meristem initials (root apex) in an area of 0.2 × 0.2 mm, was similar in cold and warm treatments (Table 3) and these meristematic cells had diameters between 8 and 10 µm. Unexpectedly, the low temperature had no significant effect on the final cell length in *Poa* and *Rumex*, but a trend to shorter cells was found in cold-treated *Ranunculus* and in *Tussilago* (Table 3). The distance-to-apex data revealed that cell elongation was strongly reduced by low temperature in all three forb species, but not significantly in the grass species (Fig. 5). Thus, in cold-treated roots, cells remained in the small (meristematic) state over a longer distance from the apex compared to warm-treated roots, but counter-expectation, reached final cell length over a shorter distance from the apex (Fig. 6). Yet, there were much fewer elongating cells in the elongation zone than in the warm-treated roots in which cells reached final cell length over a longer distance, and there were more of these elongating cells (Fig. 6).

**Table 3.** Cell density in an area of 0.2 × 0.2 mm close to the root apex and the final cell length reached in >5 mm distance from the root apex (mean ± SD). Number of replicates (from different individuals): *Rumex*: *n* = 4; *Ranunculus*: *n* = 5; *Tussilago*: *n* = 6; and *Poa*: *n* = 6. ^#^Trend for shorter cells in *Ranunculus* (*P* = 0.09) and *Tussilago* (*P* = 0.06) under cold treatment.

	*Rumex*		*Ranunculus*		*Tussilago*		*Poa*	
	Cold	Warm	Cold	Warm	Cold	Warm	Cold	Warm
Cell density close to root apex	353 ± 44	342 ± 20	332 ± 18	351 ± 15	313 ± 25	323 ± 20	400 ± 39	400 ± 18
Final cell length (mm)	0.21 ± 0.03	0.23 ± 0.03	0.18 ± 0.04^#^	0.24 ± 0.02	0.14 ± 0.02^#^	0.19 ± 0.02	0.31 ± 0.01	0.28 ± 0.04

**Figure 7.**
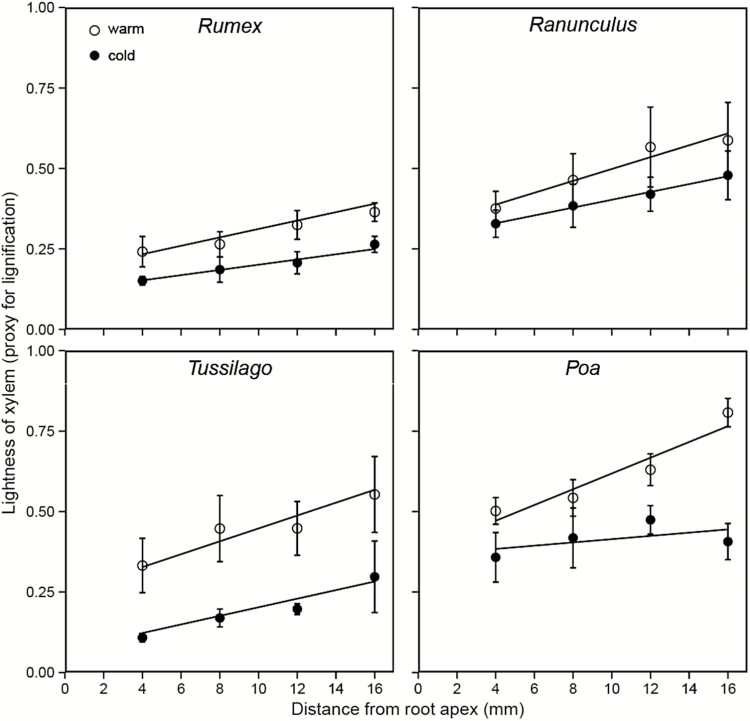
Lightness indices (mean ± SD; L-channel of the HSL colour space) as a measure for lignification of xylem elements in 4 mm steps along the terminal 20 mm of the root tips (for number of replicates, see Table 3 and for the linear models, see Table 4).

The staining with fluorescent berberine–aniline blue employed here was effective to detect xylem lignification in the thin sections of the roots. The low temperature treatment retarded the lignification of the xylem, indicating a lower rate of cell differentiation in the cold treatment. The first lignified xylem elements (fluorescent signal observed through light microscopy) emerged at a greater distance from the root apex in cold-grown roots (Fig. 5). In *Rumex* roots, the species that stopped growing at relatively warmer temperature, the lignified xylem was detectable at a similar distance from the apex in both temperature treatments. The degree of lignification, measured as lightness (L-channel value) of the fluorescent xylem, showed an overall higher sensitivity than the direct visual observation in the microscope images. The lightness values were significantly lower and remained lower with increasing distance from the apex under cold conditions for three species (trend only in *Ranunculus*; Fig. 7, Table 4). Both *Ranunculus* and *Poa* showed brighter fluorescence signals than the other two species, suggesting a higher degree of lignification compared to *Tussilago* and *Rumex* (Fig. 7; **see Supporting Information—Fig. S4**).

**Table 4.** Treatment effect on xylem lignification (L-values). Linear models for treatment (warm vs. cold) and distance from root apex (nested design; model for each species).

Species	Factor	Estimate	SE	*t* value	*P* value
*Rumex*	Intercept	0.21	0.03	6.6	<0.001
	Treatment	0.12	0.02	2.9	<0.01
	Distance (treatment)	0.01	0.01	3.4	<0.01
*R* ^2^ = 0.46					
*Ranuculus*	Intercept	0.35	0.07	5.1	<0.001
	Treatment	0.25	0.05	4.3	0.076
	Distance (treatment)	0.02	0.01	2.7	0.014
*R* ^2^ = 0.27					
*Tussilago*	Intercept	0.29	0.07	4.3	<0.001
	Treatment	0.04	0.05	0	<0.001
	Distance (treatment)	0.02	0.01	2.8	0.011
*R* ^2^ = 0.57					
*Poa*	Intercept	0.46	0.06	8.4	<0.001
	Treatment	0.26	0.04	3.7	<0.001
	Distance (treatment)	0.02	0.01	3.2	<0.01
*R* ^2^ = 0.46					

## Discussion

We grew four alpine taxa under typical alpine climate for above-ground plant tissues but at two tightly controlled, contrasting root zone temperatures. Temperature variability in the top 30 mm was substantial due to solar radiation but below that level, temperatures decreased sharply with increasing soil depth in the cold treatment. At soil depths between −40 and −60 mm, where most cold-treated roots reached the cylinder walls, temperatures were mainly below 5 °C **[see Supporting Information—Figs S1–S3]**. Lowest tip root temperatures during RER measurements were 0.7 to 1.2 °C and roots of all four alpine species were still capable to elongate at these low temperatures.

To delineate the physiological minimum temperature for root growth (*sensu*: root formation), we took the single deepest root per species formed during the experiment and that root position corresponded to 1.0 °C in *P. alpina* and between 1.0 and 1.2 °C for the three forb species taking the hourly maximum temperature during the period of the single deepest root formation into account, the critically low temperature for root growth is 1.4 °C (Table 1).

Given that one single deepest root may not fully represent the minimum temperature threshold for the species, we calculated a mean of >20 single deepest roots per species and their corresponding root temperatures. *Poa* had the lowest mean temperature threshold with 1.5 °C, followed by 1.9 °C in *T. farfara*, 2.0 °C in *R. glacialis* and 2.4 °C in *R. alpinus.* However, the hourly maximum temperatures for this mean per species covered a much higher temperature range since not all deepest roots per cylinder reached the depth beyond −60 mm where temperature fluctuations became small (Table 1).

The temperatures thresholds for root growth observed here are slightly lower than those reported for montane tree taxa (Schenker *et al.* 2014), arctic plants (Bliss 1956; Ellis and Kummerow 1982) and our previous estimates. These were based on longer root observation intervals (4 days) for *Ranunculus* and *Poa* (Nagelmüller *et al.* 2016a), which arrived at thresholds close to 2 °C for these two species.

The root systems that developed in the cold soil profiles were highly retarded and also AGB was negatively affected compared to controls, except for *R. glacialis*, showing reduced leaf area but not significantly lowered leaf mass. Biomass allocation expressed as SMF and RMF was reduced under the cold treatment, except LMF. Unexpectedly, SMF was significantly higher in *Ranunculus* and *Tussilago* under the cold treatment. The higher SMF could be an effect of delayed leaf unfolding, causing petiole mass to contribute to the higher stem fractions (in terms of their function, petioles were considered to belong to the stem fraction). Compared to the earlier *in situ* experiment with the species *R. glacialis* and *P. alpina* (Nagelmüller *et al.* 2016a), the low temperature treatment was more severe here, which contributed to the significantly negative effects on AGB that was not observed in that earlier work.

It is a crucial issue in studies that aim at defining thermal thresholds for growth that temperatures do not fluctuate and temperature sensors embody the needed temporal and spatial resolution as well as the accuracy. Temperature means may be fully misleading, particularly, if they include periods with higher temperatures that might be sufficient for plants to grow. In the present study, we always considered the hourly maximum temperatures during the corresponding observation period (RER and root formation period, respectively).

Counter-expectation, the cell elongation zone in root tips was not longer but shorter in cold-grown roots as we measured a shorter distance from the root apex to the first fully elongated cells (Fig. 6). We explain this observation by the fact that the sharp temperature gradient in the cold treatment did not permit root tips to expand beyond a certain temperature. Similar observations emerged in *Arabidopsis thaliana* roots exposed to 4 °C: root apices were deformed and growth zones shortened, causing a swelling in the primary roots (Plohovska *et al.* 2016). These authors explained the negative effect of low temperature on root elongation to be associated with impaired organization of the cytoskeleton, particularly microfilaments. Also in an earlier study on roots of three cultivars of winter wheat which differed in frost tolerance, Abdrakhamanova *et al.* (2003) related the changes in the microtubuli organization (especially, the disassembling of microtubuli during frost events) to the capability of roots of the tolerant cultivar to recover from frost and to grow at 4 °C.

We also assume feedback regulation from the elongation zone to the meristematic zone that causes a cessation of further cell production at such minimum temperature well known from root growth kinematic studies (Silk 1992; Beemster and Baskin 1998; Rost 2011; Kumpf and Nowack 2015). Interestingly, the most low temperature tolerant species in terms of root elongation and rooting depth (and the only monocot), *P. alpina*, almost retained the proportion between the length of the cell elongation zone and the meristematic zone.

The higher sensitivity of cell elongation compared to cell division at extremely low positive temperature causes the pressure for root tip progression into deeper (and colder) soils to cease and thus reduces RER close to zero. The temperature limit for cell elongation can be expected to occur at slightly higher than the minimum temperatures estimated from root tip position (Table 1) because the end of the elongation zone was between 2 and 4 mm above the tip, corresponding to ca. 0.1–0.2 K higher temperature. While cell elongation was clearly limited, the root apical meristem was still able to produce new cells, which accumulated for a longer distance from the apex and stayed small (in meristematic size). In the warm treatment, root cells at a similar distance from the apex kept elongating to final cell length (Fig. 6). These findings are in line with results of earlier studies that showed that growth restriction at low temperature does not start with an inhibition of cell division and cell production (Francis and Barlow 1988; Körner and Pelaez Menendez-Riedl 1989). Yet, at some point (in time and/or space), cell production must be down-regulated through feedback from limited cell elongation and cell differentiation.

We assume that the limitation of cell enlargement and the associated cell differentiation process are critical for root growth at very low temperature. Xylem differentiation may play an important role and lignification is a potentially critical candidate. The lignification of conduits is essential for the functionality of the xylem, and only a tight conduit system can contribute to the needed turgor pressure required for soil penetration. In the low temperature treatment, lignified, and thus functional xylem vessels, became visible only at a greater distance from the root apex, and the lignin signal was less intense, although these cells had more time (in the sense of tissue development) to accumulate lignin in the cell walls. The limited lignification of cold-grown roots may contribute to the fragility and overall ‘glassy’ texture of the whitish root tissues produced below 5 °C. Similar morphological changes in cold-treated roots were reported by Schenker *et al.* (2014) and Nagelmüller *et al.* (2016a). However, the biochemical processes underlying the inhibited lignification are still unclear, especially, whether the synthesis of lignin and its precursors, and/or the lignin deposition (polymerization) are affected under cold temperature, awaits a further explanation. Cold acclimation in plants (to chilling, positive temperatures) has often been associated with increased lignin contents in different plant organs including roots (Cabane *et al.* 2012 and citations therein). On the other hand, Donaldson (2001) reported that lignification of the secondary wall of latewood tracheids was often incomplete at the onset of winter, thus suggesting that lignification is sensitive to temperature. The lower temperature threshold for xylogenesis in the alpine *Rhododendron* shrub (2.0 ± 0.6 °C) than in conifers at the treeline (4–5 °C; Rossi *et al.* 2008) has been interpreted as a consequence of exposure to cooler microclimate of the alpine shrub (Li *et al.* 2016), particularly, at nights when radiation losses are high and convective heat exchange is low. The critically low temperature for lignification in the alpine herb and grass species observed here corroborated this minimum temperature and may be a common threshold for lignification in many cold-adapted angiosperms.

## Conclusions

Roots grown at temperatures between 1 and 5 °C showed strongly reduced elongation rates so that these roots contributed very little to the entire root system compared to control roots grown at 10 °C. Accordingly, total root biomass was substantially reduced and hardly any secondary roots were formed at temperatures below 5 °C. Temperatures in the range of 0.8 to 1.4 °C are critically low temperature thresholds for root formation in the four studied alpine plant species. The terminal zones of root tips exposed to such temperatures showed clearly inhibited cell elongation and xylem lignification. We conclude that cell differentiation and lignification are the crucial processes that prevent any further extension of root tips into colder soil space and limit tissue formation in cold environments.

## Sources of Funding

This study was funded by the Department of Environmental Sciences, University of Basel, and the Crop Science Group of the Polytechnical University (ETH) Zurich.

## Contributions by the Authors

S.N., E.H. and C.K. designed the research; S.N. performed the research; S.N. analysed the data; and S.N., E.H. and C.K. wrote the paper.

## Conflicts of Interest

None declared.

## Supporting Information

The following additional information is available in the online version of this article—

**Figure S1**. Frequency distribution of the hourly soil temperatures in the six sensor depths in the four baths (three cold and one warm control bath) during the 29 treatment days.

**Figure S2**. The temperature course of the seven temperature sensors in the cold treatment cylinders (bath 2) during the period of root elongation measurements (RER).

**Figure S3**. Estimation of mean root tip temperature for a RER per 12 h from soil depth and soil temperatures (based on polynomial regressions).

**Figure S4**. Snap shots of longitudinal cuts of warm- and cold-treated root tips showing the reduced lignification.

## Supplementary Material

Supporting-Information
