# Supplementary material for: Low temperature limits for root growth in alpine species are set by cell differentiation
Source: AoB Plants. 2017 Oct 19;9(6):plx054. doi: 10.1093/aobpla/plx054 (PMC5710522; doi:10.1093/aobpla/plx054)
Supplement: Supporting-Information [file plx054_suppl_supporting-information.doc]

**Supporting Information**, **Figure S1.**

Frequency distribution of the hourly soil temperatures in the six sensor depths during the 29-treatment-days (no roots at sensor depth of -140 mm, not shown). Note, the few warmer hours in cold bath 3 (red circles) caused by cooling system failure which occurred before root elongation (RER) measurements started.

**Supporting Information**, **Figure S2.**


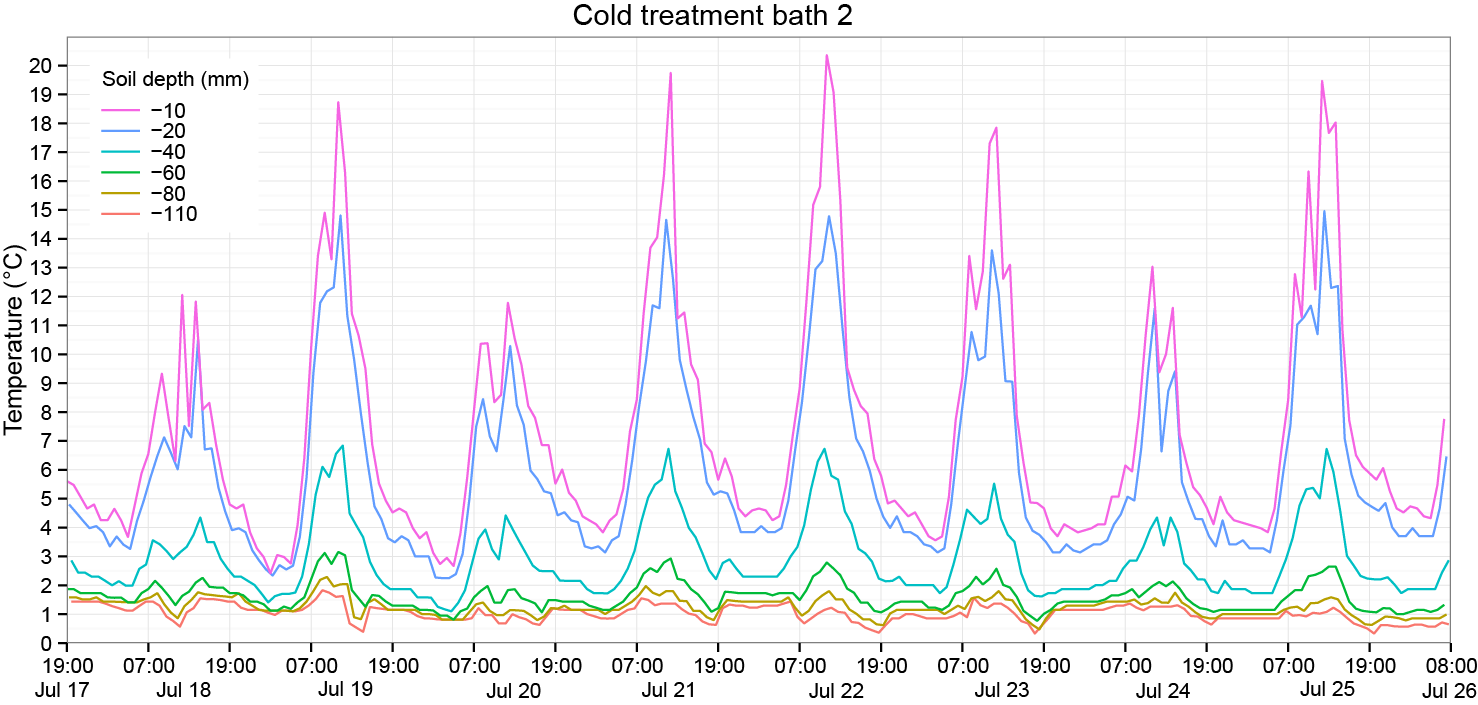


Temperature course of the seven temperature sensors in the cold treatment cylinders (bath 2) during the period of root elongation measurements (RER). Note, no roots were measured in the two uppermost (warm) soil layers of the cold bath, but these temperatures indicate that above-round plant tissues were exposed to typical alpine climate conditions (high radiation, cool nights).

**Supporting Information**, **Figure S3.**

Estimation of mean root tip temperature for a RER 12h^-1^ from soil depth and soil temperatures. Gray functions show the 12 fits for a RER 12h^-1^ based on polynomial regressions of soil temperatures (measured hourly by T resistors at seven soil depths) and soil depths. Upper graph shows an example of RER 12h^-1^ with night hours (19:00-7:00), lower graph with day hours (7:00-19:00). Red symbols show the resulting mean root tip temperature (value in parenthesis) and black bars indicate the coldest and warmest hourly temperatures for the selected RER 12h^-1^.

**Supporting Information**, **Figure S4.**


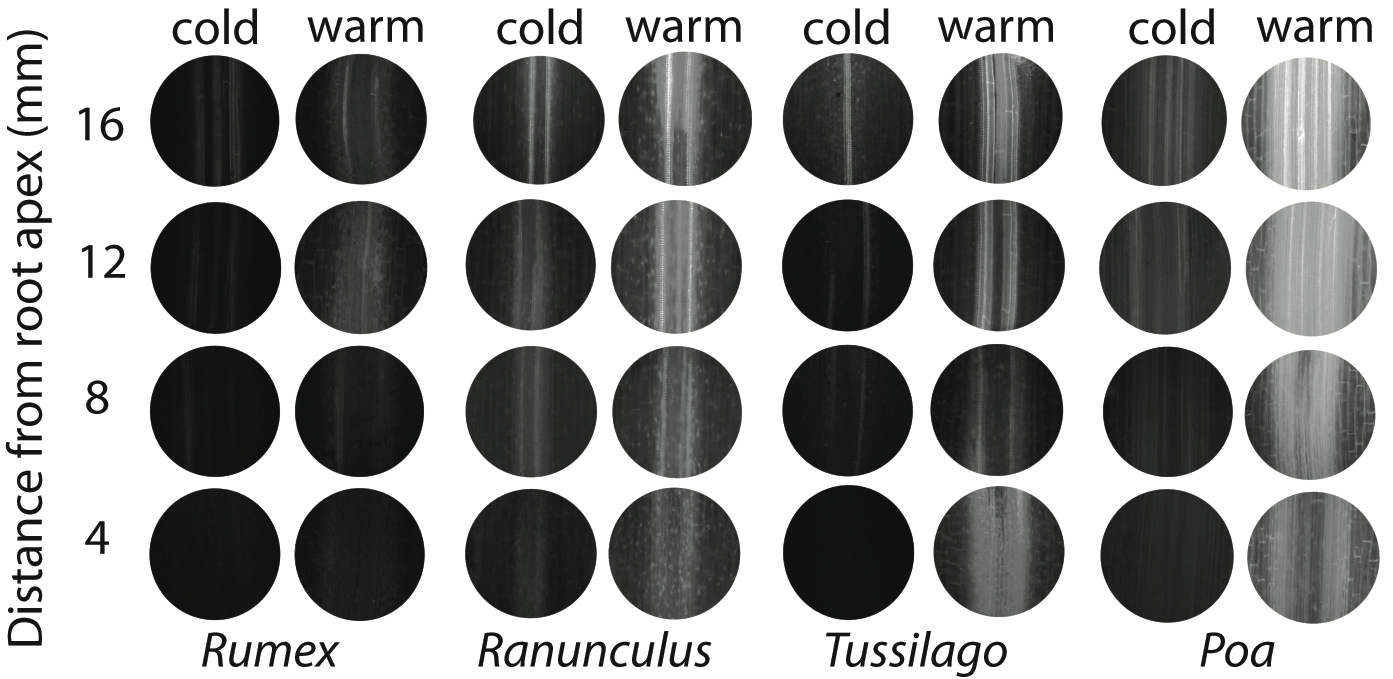


Snap shots of 0.6 mm diameter of longitudinal cuts of the central cylinder of warm and cold treated root tips. Xylem elements were stained by berberine hemi-sulfate and aniline blue. The lower intensity of the fluorescent signal in the xylem of cold treated roots indicates a reduced lignification. HSL color space images with the lightness values from the L channel were converted here to black-white images.


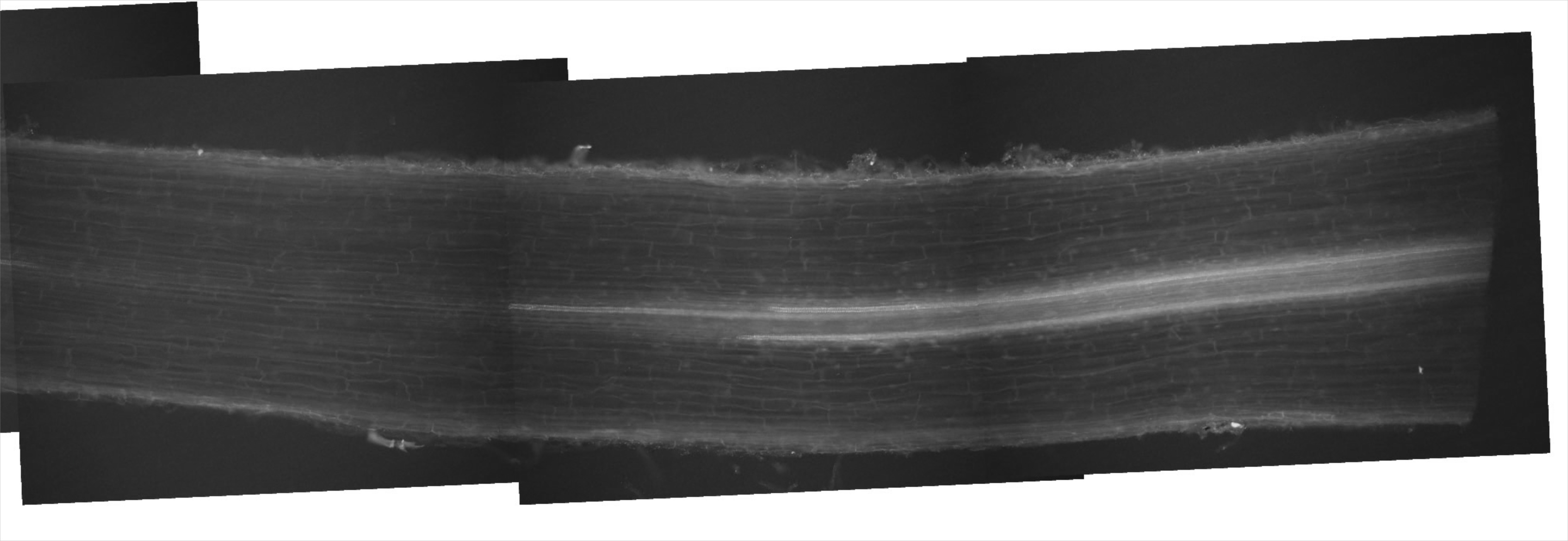


Longitudinal thin section with florescent xylem (Berberin-Hemisulfat staining) in the central cylinder. Snapshots above were taken from such images of the thin sections.
